# Supplementary material for: Altered O-linked glycosylation in benign and malignant meningiomas
Source: PeerJ. 2024 Jan 22;12:e16785. doi: 10.7717/peerj.16785 (PMC10809981; doi:10.7717/peerj.16785)
Supplement: Supplemental Information 1 [file peerj-12-16785-s001.docx]

**MIQE Checklist**

| \| **Category** \| **Item to be described/detailed** \| **Page No.** \| **Author Comments** \| \| --- \| --- \| --- \| --- \| \| **SAMPLE** \| Cell lines \| 4 \| Row 99 \| \|  \| Method of dissection/procurement \|  \|  \| \|  \| Processing procedure \|  \|  \| \|  \| If frozen, how and how quickly? \|  \|  \| \|  \| If fixed, with what and how quickly? \|  \|  \| \|  \| Storage conditions and duration \|  \|  \| \| **EXTRACTION** \| TRIzol reagent \| 6 \| Row 153 \| \|  \| Reagents/kits/modifications \| 6 \| Total RNA was extracted according to the manufacturer’s protocol. The cDNA synthesis was performed using a SensiFAST cDNA synthesizer kit. \| \|  \| DNAse or RNAse treatment \| 6 \| Total RNA was extracted according to the manufacturer’s protocol. The cDNA synthesis was performed using a SensiFAST cDNA synthesizer kit. \| \|  \| Evidence for lack of contamination (DNA or RNA) \| 6 \| Total RNA was extracted according to the manufacturer’s protocol. The cDNA synthesis was performed using a SensiFAST cDNA synthesizer kit. \| \|  \| Nucleic acid quantification \| 6 \| Total RNA was extracted according to the manufacturer’s protocol. The cDNA synthesis was performed using a SensiFAST cDNA synthesizer kit. \| \|  \| RNA integrity \| 6 \| Total RNA was extracted according to the manufacturer’s protocol. The cDNA synthesis was performed using a SensiFAST cDNA synthesizer kit. \| \| **REVERSE TRANSCRIPTION** \| SensiFAST cDNA synthesizer kit \| 6 \| Row 155 \| \|  \| RNA amount and reaction volume \|  \| The cDNA synthesis was performed using a SensiFAST cDNA synthesizer kit. \| \|  \| Priming oligo sequence(s) \|  \| The cDNA synthesis was performed using a SensiFAST cDNA synthesizer kit. \| \|  \| Cqs with and without reverse transcriptase \|  \|  \| \| **qPCR TARGET** \| HUGO gene abbreviation \|  \|  \| \|  \| Sequence accession number \|  \|  \| \|  \| Amplicon length \|  \|  \| \|  \| *In silico* specificity (BLAST) \| 16 \| Primer sets of all glycosyltransferases are listed in Table 2 \| \|  \| Location by exon/intron \|  \|  \| \|  \| Identify the splice variants amplified \|  \|  \| \|  \| All primer/probe sequences \| 16 \| listed in Table 2 \| \|  \| Location and identity of any oligonucleotide modifications \|  \|  \| \| **qPCR PROTOCOL** \| Complete reaction conditions, including all components and their concentrations \| 6 \| The gene amplification condition was performed as previously described (26)  Talabnin C, Talabnin K, Wongkham S. Enhancement of piperlongumine chemosensitivity by silencing heme oxygenase-1 expression in cholangiocarcinoma cell lines. Oncol Lett. 2020;20(3):2483-92. \| \|  \| cDNA/DNA amount and reaction volume \|  \| The gene amplification condition was performed as previously described (26)  Talabnin C, Talabnin K, Wongkham S. Enhancement of piperlongumine chemosensitivity by silencing heme oxygenase-1 expression in cholangiocarcinoma cell lines. Oncol Lett. 2020;20(3):2483-92. \| \|  \| Instrument identification and complete thermocycling parameters \|  \| The gene amplification condition was performed as previously described (26)  Talabnin C, Talabnin K, Wongkham S. Enhancement of piperlongumine chemosensitivity by silencing heme oxygenase-1 expression in cholangiocarcinoma cell lines. Oncol Lett. 2020;20(3):2483-92. \| \| **qPCR VALIDATION** \| Evidence for PCR specificity (gels, sequencing, or melting curves) \| 6 \| The gene amplification condition was performed as previously described (26)  Talabnin C, Talabnin K, Wongkham S. Enhancement of piperlongumine chemosensitivity by silencing heme oxygenase-1 expression in cholangiocarcinoma cell lines. Oncol Lett. 2020;20(3):2483-92. \| \|  \| Template inhibition data (template titrations) \|  \| The gene amplification condition was performed as previously described (26)  Talabnin C, Talabnin K, Wongkham S. Enhancement of piperlongumine chemosensitivity by silencing heme oxygenase-1 expression in cholangiocarcinoma cell lines. Oncol Lett. 2020;20(3):2483-92. \| \|  \| For SYBR Green I reactions, the Cq of the no template control \|  \|  \| \|  \| Calibration curves with slope and intercept \|  \| The gene amplification condition was performed as previously described (26)  Talabnin C, Talabnin K, Wongkham S. Enhancement of piperlongumine chemosensitivity by silencing heme oxygenase-1 expression in cholangiocarcinoma cell lines. Oncol Lett. 2020;20(3):2483-92. \| \|  \| PCR efficiency from the slope \|  \| The gene amplification condition was performed as previously described (26)  Talabnin C, Talabnin K, Wongkham S. Enhancement of piperlongumine chemosensitivity by silencing heme oxygenase-1 expression in cholangiocarcinoma cell lines. Oncol Lett. 2020;20(3):2483-92. \| \|  \| r^2^ of the calibration curve \|  \| The gene amplification condition was performed as previously described (26)  Talabnin C, Talabnin K, Wongkham S. Enhancement of piperlongumine chemosensitivity by silencing heme oxygenase-1 expression in cholangiocarcinoma cell lines. Oncol Lett. 2020;20(3):2483-92. \| \|  \| Evidence for the linear dynamic range \|  \|  \| \|  \| Evidence for the limit of detection \|  \|  \| \|  \| For multiplexed assays, the efficiency and limit of detection of each assay \|  \|  \| \| **DATA ANALYSIS** \| qPCR analysis method/software \| 6 \| Relative mRNA expression was determined using the 2Ct method (27).  Livak KJ, Schmittgen TD. Analysis of relative gene expression data using real-time quantitative PCR and the 2(-Delta Delta C(T)) Method. Methods. 2001;25(4):402-8. \| \|  \| Method of Cq determination \|  \|  \| \|  \| Results of no template controls \|  \|  \| \|  \| Justification of number and choice of reference genes \|  \|  \| \|  \| Normalization method \| 6 \| *β*-*Actin* was used as the internal control to normalize the expression of the target genes. \| \|  \| Number and stage (reverse transcription or qPCR) of technical replicates \|  \| Relative mRNA expression was determined using the 2Ct method (27).  Livak KJ, Schmittgen TD. Analysis of relative gene expression data using real-time quantitative PCR and the 2(-Delta Delta C(T)) Method. Methods. 2001;25(4):402-8. \| \|  \| Intra-assay variation in terms of concentration, not Cq \|  \|  \| \|  \| Statistical methods/software \| 7 \| All analyses were performed with GraphPad Prism software (version 8.0; GraphPad Software, Inc.). A P < 0.05 indicated a statistically significant difference. \| |
| --- | --- | --- | --- | --- | --- | --- | --- | --- | --- | --- | --- | --- | --- | --- | --- | --- | --- | --- | --- | --- | --- | --- | --- | --- | --- | --- | --- | --- | --- | --- | --- | --- | --- | --- | --- | --- | --- | --- | --- | --- | --- | --- | --- | --- | --- | --- | --- | --- | --- | --- | --- | --- | --- | --- | --- | --- | --- | --- | --- | --- | --- | --- | --- | --- | --- | --- | --- | --- | --- | --- | --- | --- | --- | --- | --- | --- | --- | --- | --- | --- | --- | --- | --- | --- | --- | --- | --- | --- | --- | --- | --- | --- | --- | --- | --- | --- | --- | --- | --- | --- | --- | --- | --- | --- | --- | --- | --- | --- | --- | --- | --- | --- | --- | --- | --- | --- | --- | --- | --- | --- | --- | --- | --- | --- | --- | --- | --- | --- | --- | --- | --- | --- | --- | --- | --- | --- | --- | --- | --- | --- | --- | --- | --- | --- | --- | --- | --- | --- | --- | --- | --- | --- | --- | --- | --- | --- | --- | --- | --- | --- | --- | --- | --- | --- | --- | --- | --- | --- | --- | --- | --- | --- | --- | --- | --- | --- | --- | --- | --- | --- |
